# Unraveling hematopoietic stem cell ontogeny through single-cell multi-omics approaches

**DOI:** 10.3389/fcell.2025.1704887

**Published:** 2025-11-21

**Authors:** Yan Jia, Sixuan Huo, Fei Wu, Ningning Song, Mei Li

**Affiliations:** School of Life Science and Technology, Shandong Second Medical University, Weifang, China

**Keywords:** hematopoietic stem cells (HSCs), single-cell multi-omics, endothelial-to-hematopoietic transition (EHT), aorta-gonad-mesonephros (AGM) region, developmental heterogeneity

## Abstract

Definitive hematopoietic stem cells (HSCs) originate *de novo* within the vertebrate aorta-gonad-mesonephros (AGM) region via endothelial-to-hematopoietic transition (EHT) from hemogenic endothelial cells (HECs). The application of single-cell multi-omics has significantly deepened our knowledge about hematopoietic development. In this review, we focus on the ontogeny of HSCs and summarize novel insights gained from single-cell omics studies. These include newly identified components of hematopoietic regulatory networks, the cellular heterogeneity during HSC generation, innovative strategies for enriching rare cell subpopulations, and newfound knowledge about the AGM microenvironment. In the concluding section, we discuss key unresolved questions related to *in vivo* generation and *in vitro* induction of HSCs, while exploring the potential of single-cell omics to propel future research in this field.

## 1 Introduction

Hematopoietic stem cells (HSCs) reside at the apex of the hematopoietic hierarchy, with the capacities of self-renewal and multilineage differentiation into all types of blood cells (Ng and Alexander, 2017; Karamitros et al., 2018; Laurenti and Göttgens, 2018). Owing to their ability to fully reconstitute the hematopoietic system, HSC transplantation has become an essential therapeutic strategy for a range of hematological diseases (e.g., leukemia, anemia) and immune disorders (Thompson et al., 2018; Wilkinson et al., 2020; Jensen et al., 2023; Kantarjian et al., 2025). Consequently, the *in vitro* generation of functional HSCs represents a highly promising research direction at present and in the future. However, due to the complexity of HSC development, our understanding of the molecular and cellular mechanisms governing HSC emergence remains incomplete. Therefore, the *in vitro* induction of sufficient numbers of HSCs with multilineage differentiation capacity remains a significant challenge (Piau et al., 2023; Ng et al., 2025).

HSCs originate from definitive hematopoiesis during embryonic development, primarily via the endothelial-to-hematopoietic transition (EHT) process in the aorta-gonad-mesonephros (AGM) region (Medvinsky and Dzierzak, 1996; Bertrand et al., 2010; Boisset et al., 2010; Kissa and Herbomel, 2010). In recent decades, studies on the emergence of HSCs *in vivo* have revealed the presence of various continuously transitioning intermediate stages, including hemogenic endothelial cells (HECs), type I pre-HSCs, and type II pre-HSCs (Zovein et al., 2008; Rybtsov et al., 2011; Rybtsov et al., 2014). Within the specialized AGM microenvironment, a complex regulatory network comprising both intrinsic and extrinsic factors orchestrates HEC specification and the EHT process. Key transcription factors, such as GATA2, RUNX1, and GFI1/GFI1B, play critical roles by collaboratively suppressing the expression of endothelial-related genes while activating hematopoietic programs, thereby facilitating the fate transition from endothelial to hematopoietic cells (Tober et al., 2016). Concurrently, signaling pathways including Notch, Wnt/β-catenin, and BMP critically contribute to this process (Kumano et al., 2003; Gama-Norton et al., 2015; Richter et al., 2017; Canu and Ruhrberg, 2021). However, the formation of HSCs involves a precise progression through distinct cellular states, yielding rare and transient intermediates, including HECs and pre-HSCs. This complexity has posed significant challenges for research on HSC generation *in vivo*.

In recent years, the emergence of single-cell sequencing technologies has provided novel approaches for studying embryonic hematopoietic development. In this review, we discuss how these single-cell technologies have contributed to identifying new molecular regulators of HSC emergence, resolving the cellular heterogeneity during the transition from vascular to hematopoietic cells and providing higher resolution of the AGM microenvironment. Finally, we discuss the unresolved questions in hematopoietic development and *in vitro* generation, highlighting the potential of single-cell sequencing technologies in driving future research.

## 2 Multiple waves of embryonic hematopoietic development

**FIGURE 1:**
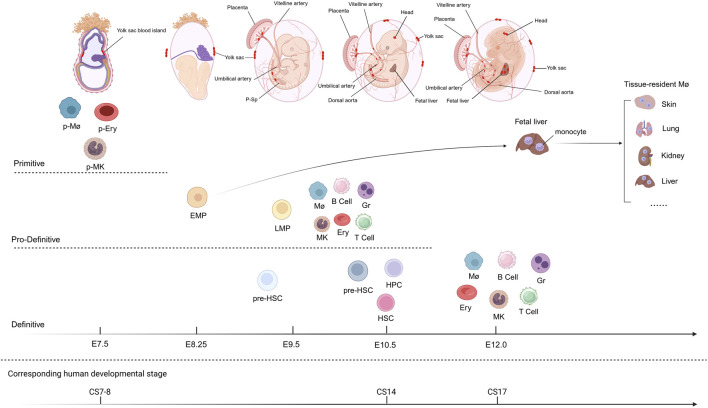
Ontogeny of the embryonic hematopoiesis. Hematopoiesis in mice occurs in three sequential waves: primitive, pro-definitive, and definitive. The primitive wave originates in the yolk sac (YS) at embryonic day (E) 7.5 and produces primitive erythrocytes (p-Erys), primitive macrophages (p-Møs), and primitive megakaryocytes (p-MKs). The pro-definitive wave arises primarily from the YS at E8.25 and generates erythro-myeloid progenitors (EMPs). The EMPs migrate to the fetal liver and differentiate into erythrocytes (Erys), megakaryocytes (MKs), macrophages (Møs), granulocytes (Grs), T cells, and B cells. Tissue-resident macrophages (Tissue-resident Møs) in several adult tissues are derived from this wave. This wave also produces lymphomyeloid progenitors (LMPs). The definitive wave gives rise to hematopoietic stem cells (HSCs) primarily in the aorta-gonad-mesonephros (AGM) region at E10.5, which further migrate to the fetal liver to expand and differentiate into multiple hematopoietic lineages, including Ery, MKs, Mø, Gr, T cells and B cells. HSCs finally migrate to the bone marrow (BM) and support life-long hematopoiesis, not shown here. Definitive HSCs are also produced in the placenta, YS, vitelline artery (VA) and umbilical artery (UA). Hematopoietic sites at different developmental stages are indicated by red dots. The sequence and timing of human hematopoietic ontogeny are presented in the bottom panel.

Embryonic hematopoietic development is generally categorized into three sequential waves according to their timing and the types of cells generated (Orkin and Zon, 2008; Kauts et al., 2016). A summary of the three waves of hematopoiesis in mice and human is presented in Figure 1. In the mouse embryo, the first wave of primitive hematopoiesis is initiated in the yolk sac (YS) blood islands at embryonic day (E) 7.5, producing primitive erythrocytes, macrophages, and megakaryocytes (Palis et al., 1999; Xu et al., 2001; Tober et al., 2007; Palis and Yoder, 2001). The primitive erythrocytes produced during this period are characterized by their short lifespan and the expression of embryonic hemoglobin, which meet the need for oxygen transport in the early embryo (McGrath et al., 2008; Palis, 2014; Palis and Yoder, 2001). While the emergence of primitive erythrocytes is independent of RUNX1, their subsequent maturation critically requires this transcription factor (Yokomizo et al., 2008). In humans, primitive hematopoiesis that occurs independently of HSCs is also observed, specifically in Carnegie stages (CS) 7-8 within the YS blood islands, resulting in the formation of primitive erythrocytes, macrophages, and megakaryocytes (Ivanovs et al., 2017; Hislop et al., 2024). Furthermore, primitive macrophages serve as precursors of adult microglia in both the human and mouse brain (Ginhoux et al., 2010; Bian et al., 2020).

The second wave of hematopoiesis primarily arises from the YS at E8.25 and is also observed in the placenta and umbilical artery (UA), collectively referred to as pro-definitive hematopoiesis (Ciau-Uitz et al., 2014; Tober et al., 2016; Plein et al., 2018). During this period, erythroid-myeloid progenitors (EMPs) with the capacity for erythroid and myeloid differentiation are generated from HECs in the YS via a RUNX1-dependent process and are characterized by the immunophenotype KIT^+^CD41^+^. These progenitors further become KIT^hi^CD41^+^CD16/32^+^, distinguishing them from primitive hematopoiesis. Although EMPs do not possess long-term hematopoietic capacity, they can transiently generate adult-like red blood cells following transplantation (Tober et al., 2013; McGrath et al., 2015). With the exception of brain microglia, most adult tissue-resident macrophages are derived from this wave of hematopoiesis. MYB^+^ EMPs from the YS give rise to monocytes in the fetal liver (FL), which subsequently differentiate into tissue-resident macrophages in organs such as the skin, liver, kidney, and lung (Hoeffel et al., 2015). However, whether YS-derived EMPs contribute to the formation of adult vasculature in organs remains controversial, potentially due to differences in the transgenic mouse strategies employed (Plein et al., 2018; Feng et al., 2020). Interestingly, Williamson et al. identified a population of clonogenic endothelial-macrophage (EndoMac) progenitors within adult aortic vessels. These cells exhibit bidirectional potential to differentiate into both endothelial cells (ECs) and macrophages. Originating from the embryonic YS, these progenitors bypass the FL to form monocytes and display molecular characteristics intermediate between EMPs and CX3CR1^+^ pre-macrophages (Williamson et al., 2024). The second wave also includes lymphomyeloid progenitors that occur in the E9.5 YS and para-aortic splanchnopleura (P-Sp), capable of differentiating into granulocyte-macrophage (GM), T cells, and B cells (Böiers et al., 2013; Lin et al., 2014). In addition, pre-HSCs capable of directly engrafting neonates but not adults are generated in the YS and P-Sp at E9.0 in this wave. These cells subsequently acquire the capacity to reconstitute adult hematopoiesis upon secondary transplantation (Yoder et al., 1997a; Yoder et al., 1997b). This is likely because these early cells require the unique embryonic hematopoietic microenvironment, particularly the FL, for engraftment and maturation. In humans, EMPs are identified in the YS during CS 7. They have also been identified in the placenta, referred to as placental EMPs (PEMPs). Both populations share similar transcriptional profiles and cluster computationally with mouse YS EMPs rather than with HSCs from the AGM region or FL (Tyser et al., 2021; Thomas et al., 2023).

The third wave of hematopoiesis, known as definitive hematopoiesis, produces HSCs with multilineage differentiation potential. In mice, the first HSCs emerge in the AGM region at E10.5 (Medvinsky and Dzierzak, 1996; Zhou et al., 2019a). HSCs are also thought to be produced in the UA, vitelline artery (VA), YS, placenta, and head (Kumaravelu et al., 2002; Ottersbach and Dzierzak, 2005; Li Z. et al., 2012; Gordon-Keylock et al., 2013). During this period, a specialized type of ECs known as HECs buds off through EHT and aggregates to form intra-aortic hematopoietic clusters (IAHCs) within the vascular lumen (Boisset et al., 2010; 2015). Approximately 500–700 IAHCs (CD31^+^KIT^+^) are formed in the AGM region of E10.5–E11.5 mouse embryos, containing only 1–2 HSCs (Yokomizo and Dzierzak, 2010; Vink and Dzierzak, 2023). Actually, IAHCs are primarily composed of hematopoietic progenitor cells (HPCs) and pre-HSCs, including CD45^−^ type I and CD45^+^ type II pre-HSCs (Taoudi et al., 2008; Rybtsov et al., 2011; Vink and Dzierzak, 2023). It has been found that IAHCs are predominantly located in the mid-section of the aorta (around the junction of the aorta and the VA) (Yokomizo and Dzierzak, 2010). Furthermore, most IAHCs are found in the ventral wall of dorsal aorta, which may be attributed to variations in Notch activity. High Notch activity in the dorsal region supports the maintenance of endothelial characteristics, while low Notch activity in the ventral region favors the formation of HSCs (Gama-Norton et al., 2015). Additionally, the polarity of HSC emergence is also regulated by BMP signaling (Durand et al., 2007; Wilkinson et al., 2009). Subsequently, pre-HSCs migrate through the bloodstream to the FL, where they further mature into HSCs and differentiate, gradually replacing those cells derived from EMPs (Canu and Ruhrberg, 2021; Yokomizo and Suda, 2024). The FL has been considered the principal site of HSC expansion (Ema and Nakauchi, 2000). However, recent multi-color lineage tracing conducted by Ganuza et al. revealed that the FL contributes only approximately a two-fold expansion to adult HSCs (Ganuza et al., 2022a; Ganuza et al., 2022b). Finally, HSCs colonize the bone marrow (BM) (around E15.5), where they maintain lifelong hematopoiesis in adults (Mikkola and Orkin, 2006; Dzierzak and Bigas, 2018). In humans, HSCs likewise originate in the AGM region, with the earliest detection reported at CS14 (Ivanovs et al., 2011).

In recent years, several studies combing cellular barcoding/lineage tracing with single-cell RNA sequencing (scRNA-seq) have renewed our insights into the contribution of HSCs and hematopoietic progenitors to the embryonic and young adult hematopoietic system. Ulloa et al. performed scRNA-seq of zebrafish hematopoietic stem and progenitor cells (HSPCs) and transcriptionally identified HSC-independent progenitors by inferring developmental lineage trajectories. Subsequent lineage-tracing experiments using a *drl:CreERT*2 zebrafish model demonstrated that embryonic hematopoiesis is primarily sustained by HSC-independent progenitors, whose contribution far exceeds that of HSCs (Ulloa et al., 2021). Corroborating this finding, Dignum et al. using *in vitro* clonal assays, found that embryonic HSCs and multipotent progenitors (MPPs) independently originate from distinct HEC populations. Moreover, they identified CXCR4 as a marker to distinguish HSC-competent HECs from MPP-competent HECs (Dignum et al., 2021). Recent study by Yokomizo et al. discovered that HLF^+^KIT^+^ pre-HSPCs can independently give rise to both HSCs and various progenitors simultaneously, with EVI1^hi^ pre-HSPCs being more biased toward HSC formation. Using an *Evi1-CreERT2* mouse model to trace HSCs, they found that HSCs rarely differentiate into progenitors and contribute minimally to embryonic hematopoiesis, suggesting that embryonic HSCs may primarily serve as a reservoir for adult hematopoiesis (Yokomizo et al., 2022). Patel et al. employing a Sleeping Beauty (SB)-based cellular barcoding system and *Flt3-CreER* mice-mediated fate mapping, identified and tracked a population of HSC-independent embryonic multipotent progenitors (eMPPs). They found that these eMPPs, likely descendants of pre-HSCs, predominantly contribute to hematopoiesis in embryos and young adults and serve as the major source of lymphoid cells (Patel et al., 2022). Complementing these findings, Kobayashi et al. used *Fgd5-CreERT2* and *Cdh5-CreERT2* mice to trace HSC and EC outputs, respectively. They identified multiple waves of endothelial-derived lymphoid progenitors that constitute a significant proportion of T and B cells in adult mice, with B-1a cells being almost entirely derived from HSC-independent hematopoiesis (Kobayashi et al., 2023). Collectively, these studies clarify the respective contributions of HSCs and HSC-independent progenitors to embryonic and adult hematopoiesis and revise the previously HSC-centric hierarchy of hematopoiesis, providing new insights for guiding the *in vitro* induction of HSCs from human pluripotent stem cells (hPSCs).

## 3 Applications of single-cell sequencing in elucidating HSC emergence in model animals

**FIGURE 2:**
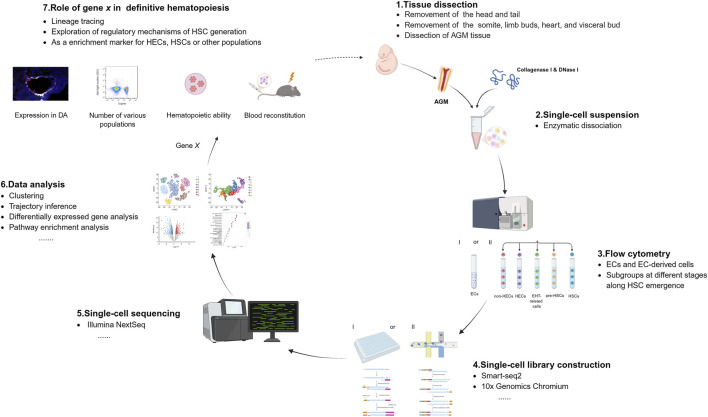
Experimental strategy to generate scRNA-seq data from embryonic hematopoietic tissues aorta-gonad-mesonephros (AGM) tissue is dissected from mouse embryos and enzymatically dissociated into single-cell suspensions, followed by flow cytometry to isolate cells in two experimental models: I. Sorting all endothelial cells (ECs) and EC-derived hematopoietic populations; II. Sorting of distinct cell populations at different stages of hematopoietic stem cell (HSC) emergence. Single-cell libraries are then constructed using Smart-seq2 and 10x Genomics, followed by sequencing and bioinformatic analyses including trajectory inference, differential gene expression, pathway enrichment, and other analyses. Gene *X* represents a novel regulator identified through single-cell sequencing. Its functional role in HSC emergence can be experimentally validated by assessing its effects on HSC number, hematopoietic potential, and transplantation reconstitution capacity, thereby elucidating its involvement in HSC ontogeny.

The application of single-cell sequencing technologies have greatly advanced our understanding of the molecular regulatory networks, cellular heterogeneity, and microenvironmental regulation during HSC ontogeny. In Figure 2, we summarize the experimental strategy to generate scRNA-seq data from mouse embryonic hematopoietic tissues. In the following sections, we review key contributions of single-cell omics to revealing the highly dynamic process and regulatory mechanisms underlying HSC development in model animals.

### 3.1 Uncovering novel regulators in the molecular network underlying HSC development

#### 3.1.1 Transcriptional regulators

The emergence of HSCs is orchestrated by a complex transcriptional regulatory network. RUNX1 (core binding factor α, CBFα) is the first identified specific marker for HECs, enhancing DNA-binding affinity through interaction with CBFβ. Absence of *Runx1* results in impaired EHT and failure of definitive hematopoiesis (Tober et al., 2016; North et al., 1999; Li et al., 2006; Chen et al., 2009). GFI1 and GFI1B have been identified as direct downstream targets of RUNX1 (Lancrin et al., 2012). Previous studies have indicated that GFI1 expression is initiated in HECs, while GFI1B is expressed in IAHCs. Both transcription factors facilitate HSC emergence by suppressing the expression of endothelial-specific genes in HECs (Thambyrajah et al., 2016). GATA2 has also been shown to play an essential role in EHT by inhibiting the expression of endothelium-associated genes via GFI1B during HSPC maturation, and its loss leads to abnormal apoptosis of HSCs (De Pater et al., 2013; Kang et al., 2018). Additionally, GATA3, predominantly expressed in HECs and early HSC precursors, has also been identified as a critical transcription factor for HSC generation (Zaidan et al., 2022).

In recent years, the emergence of single-cell transcriptomics has revolutionized our understanding of the dynamic expression patterns of key transcription factors. The cell fate transitions from ECs to HECs and then to HSCs can be tracked to reveal the underlying regulatory mechanisms at the single-cell level. Baron et al. were among the first to use single-cell transcriptomics to investigate the *in vivo* emergence of HSCs. By profiling aortic non-HECs, HECs, and cells undergoing EHT, they uncovered the transcriptional continuum from ECs to IAHCs, identifying 88 and 127 transcription factors at E10 and E11, respectively. Hierarchical clustering grouped these transcription factors into three sequential clusters at both E10 and E11: cluster I (e.g., *Sox17*, *Hey2*, *Mecom*) active in ECs and HECs, cluster II (e.g., *Gfi1*, *Sfpi1*, *Runx1*) driving the EHT and pre-HSC specification, and cluster III (e.g., *Zfpm1*) upregulated in maturing pre-HSCs and progenitors. They also constructed transcriptional regulatory networks and found strong correlations within these networks between developmental stages, confirming that hematopoiesis is a progressive process governed by a tightly orchestrated transcriptional cascade (Baron et al., 2018).

The application of single-cell sequencing technologies has enabled the identification of multiple novel transcription factors involved in HSC generation. Cellular Indexing of Transcriptomes and Epitopes by Sequencing (CITE-seq) was employed to resolve the heterogeneity of CD31^+^ cells in the AGM region into distinct clusters. The transcription factor Meis Homeobox 1 (MEIS1) was observed to be upregulated in pre-HE cells and expressed earlier than RUNX1. Functionally, MEIS1 induces arterial endothelial cells (AECs) to differentiate into pre-HE cells during the early EHT stage but is dispensable for the subsequent transition from pre-HE cells to hematopoietic cells (Coulombe et al., 2023). Although identified as a nuclear transcriptional regulator, the function of Nuclear Protein 1 (NUPR1) in embryonic HSC emergence was previously uncharacterized. Recent scRNA-seq revealed specific expression of NUPR1 in HECs. Genetic deletion of *Nupr1* was found to accelerate HEC specification and the EHT process, resulting in a significant increase in the number of HECs and HSPCs in the AGM region, associated with elevated Tumor Necrosis Factor-α (TNF-α) expression (Hou et al., 2020; Wang et al., 2023). Previous studies demonstrated that Hepatic Leukemia Factor (HLF) is specifically expressed in IAHCs within the AGM region and in FL HSCs, with expression upregulated during HSC maturation (Yokomizo et al., 2019). Based on scRNA-seq analysis and flow cytometry analyses, HLF expression was detected in both type I and type II pre-HSCs. Surprisingly, only *Hlf*-tdTomato^+^ cells in type II pre-HSCs exhibited multi-lineage reconstitution capacity upon transplantation, in contrast to their *Hlf*-tdTomato^-^ counterparts, revealing the necessity for pre-HSCs to express HLF prior to or together with CD45 (Tang et al., 2021). However, the molecular mechanisms by which HLF regulates the maturation of type I into type II pre-HSCs require further investigation.

#### 3.1.2 Signaling pathways

In recent years, various signaling pathways have been identified that regulate the production of HSCs in the AGM region. Among them, Notch, Wnt, and BMP pathways have been demonstrated to play crucial roles in HSC emergence (Staal and Luis, 2010; Bigas and Espinosa, 2012; Kim et al., 2015). In mammals, the Notch pathway comprises multiple receptors (NOTCH1-4) and ligands (including Delta-like and Jagged families) (Bray, 2016). *Notch1* is expressed on hematopoietic cells, and its activation promotes the transition of HECs to HSCs cell-autonomously (Kumano et al., 2003). Different Notch ligands drive ECs toward distinct cell fates through the activation of Notch signaling at varying intensities. While DLL4 is critical for AEC specification, JAG1-mediated activation of Notch is indispensable for hematopoietic development. This activation induces low Notch signaling activity in AECs, which suppresses endothelial-specific gene expression, and upregulates hematopoietic genes, including *Kit*, *Itga2b*, *Runx1*, and *Gata2*, thereby driving definitive hematopoiesis in the AGM region (Gama-Norton et al., 2015; Robert-Moreno et al., 2008). The Wnt/β-catenin signaling is also essential for HSC formation in the AGM region. Previous studies have revealed β-catenin expression in AECs at the base of KIT^+^ hematopoietic clusters, indicating that Wnt/β-catenin signaling is required for the specification of HECs. However, this requirement is transient, as Wnt activity needs to be downregulated during later stages of EHT (Ruiz-Herguido et al., 2012). Moreover, Chanda et al. demonstrated in mice that Retinoic Acid (RA) signaling, mediated by RA receptor (RAR)-α, is essential for HSC emergence in the AGM region and functions through transient suppression of the Wnt/β-catenin pathway (Chanda et al., 2013). The BMP signaling pathway also plays an indispensable role in orchestrating HSC emergence within the AGM. BMP4, predominantly produced by ventral mesenchymal cells adjacent to the dorsal aorta, guides AECs toward a hematopoietic fate by upregulating key regulators such as RUNX1 and GATA2, while repressing arterial endothelial programs, thereby facilitating the EHT process (Durand et al., 2007; Wilkinson et al., 2009).

In addition to the signaling pathways described above, various other signaling pathways are involved in regulating the emergence of HSCs. In zebrafish, Sonic Hedgehog (SHH) signaling from the embryonic midline governs definitive HSC formation by promoting the migration and arterial specification of Flk1^+^ dorsal aorta precursors through the induction of vascular endothelial growth factor (VEGF) and Notch signaling, thereby establishing the vascular niche for Runx1^+^ HSC generation (Gering and Patient, 2005). Furthermore, Calcitonin receptor-like receptor (*Crlr*) also acts in this pathway downstream of SHH to directly regulate VEGF expression, as a key mediator of arterial endothelial differentiation (Nicoli et al., 2008). KIT signaling also represents an essential regulatory mechanism during HSC development. The KIT receptor on the cell surface is activated by binding of its ligand, KIT ligand (KITL, also known as Stem cell factor, SCF). KITL/KIT pathway is a key driver of the stepwise maturation from pro-HSCs to definitive HSCs. It is essential for the maturation of pro-HSCs, type I pre-HSCs and type II pre-HSCs, while type II pre-HSCs can also respond to IL-3 (Rybtsov et al., 2014). Moreover, KITL is indispensable for the survival of type II pre-HSCs and HSCs in the AGM (Azzoni et al., 2018). Inflammatory signaling pathways also plays an essential role during HSPC emergence, as evidenced by severely reduced HSPC numbers in the AGM region of interferon (IFN)-deficient mouse and zebrafish models (Li et al., 2014). These findings further highlight the complexity of signaling requirements during HSC development.

During the emergence of HSCs from AECs, the intermediates, including HECs and pre-HSCs, are scarce and quickly transitioning. Therefore, single-cell sequencing is essential for further deciphering precise changes in signaling pathway requirements during HSC emergence and for guiding the induction of HSCs *in vitro*. In the mouse vascular cell line bEND.3 co-culture system, E10.5 HECs can be induced to form HSCs, whereas E9.5 HECs cannot. scRNA-seq of the mouse E9.5 P-Sp region revealed that HECs at this stage express BMP receptors and downstream target genes, indicating activation of the BMP signaling pathway. Based on these results, the addition of exogenous BMP4 enabled the induction of E9.5 HECs into HSCs in the bEND.3 co-culture system. This finding reveals a stage-specific dependency on BMP4: it is essential for the transition of E9.5 early HECs into E10.5 late HECs but becomes dispensable thereafter for pre-HSC formation (Tsuruda et al., 2024). Mitogen-Activated Protein Kinase Kinase Kinase 3 (MEKK3), a key member of the MAP kinase kinase kinase (MAP3K) family, together with the downstream transcription factors KLF2 and KLF4, mediates the sensing and transmission of blood flow shear stress and inflammatory signals in vascular endothelial cells (Zhou et al., 2015; Cullere et al., 2015; Fisher et al., 2015; Zhou Z. et al., 2016). EC-specific MEKK3 deficiency results in hematopoietic defects in the AGM region. Single-cell sequencing revealed that pre-HE cells in E10.0 ECs MEKK3^KO^ showed significant changes in gene expression, including Notch and Wnt signaling pathways essential for HEC specification, and the transition from pre-HE cells to HECs and IAHCs was impaired. It was later revealed that MEKK3-KLF2/4 mediates the inflammatory and hemodynamic stimulation necessary for EHT (Yang et al., 2022).

Single-cell sequencing has also been used to identify novel signaling pathways that regulate HSC emergence. scRNA-seq analysis demonstrated that mechanistic Target of Rapamycin (mTOR) signaling is enriched in type I pre-HSCs compared with ECs. Endothelial-specific knockout of *Rictor*, a core component of the mTOR Complex 2 (mTORC2), was shown to be critical for HSC emergence from ECs, while it appears less essential in subsequent hematopoietic populations (Zhou F. et al., 2016). However, it remains unclear why RICTOR deficiency does not concurrently affect HPC generation. Further investigation is required to determine whether the potential to form HSCs or HPCs is predetermined in ECs or HECs.

**FIGURE 3:**
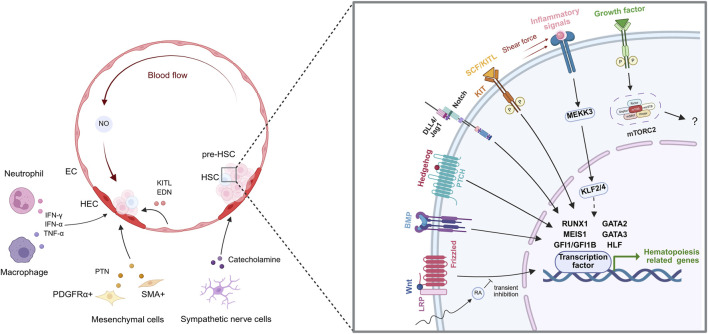
The regulation of HSC emergence by signaling pathways and the AGM microenvironment. The development of hematopoietic stem cells (HSCs) in the aorta-gonad-mesonephros (AGM) is orchestrated by multiple signaling pathways and microenvironmental regulation. We have illustrated various signaling pathways in the figure, including Wnt, BMP, Notch, Sonic Hedgehog (SHH), KIT, Mitogen-Activated Protein Kinase (MAPK), and mechanistic Target of Rapamycin (mTOR) signaling, which collaboratively promote the hematopoietic program. Meanwhile, HSC development is modulated by a complex microenvironment, comprising arterial endothelial cells (AECs), mesenchymal cells, sympathetic neural cells, immune factors, and hemodynamic shear stress, which collectively achieve precise regulation of AGM HSC ontogeny.

Altogether, the emergence of HSCs in embryos requires the coordinated regulation of multiple signaling pathways (as summarized in Figure 3). Different pathways operate at distinct developmental stages, and some must be downregulated during certain periods to ensure proper HSC production. However, the optimal use of different signaling pathway activators *in vitro*-including the timing, duration, and context of their application-remains to be determined.

#### 3.1.3 Epigenetic regulation

Epigenetic regulations, primarily including chromatin modifications and RNA modifications, play pivotal roles in HSC generation. For example, the transcription factor RUNX1 directly regulates GFI1 and GFI1B, which recruit Lysine-specific demethylase 1 (LSD1) to suppress endothelial-related genes (Thambyrajah et al., 2016). A previous study in zebrafish demonstrated that chromatin modifications modulate the BMP/SMAD signaling pathway. SMAD1/5 recruits histone deacetylase 1 (HDAC1) to the *erk* promoter region to regulate its expression, thereby preventing cells from adopting an arterial endothelial fate and promoting EHT (Zhang et al., 2014). In recent years, the role of *N* ^6^-methyl-adenosine (m6A) in HSC emergence has been clarified. Knockdown of m6A ‘writer’, *Mettl3*, impairs definitive hematopoiesis in zebrafish embryos. This impairment occurs because reduced METTL3 expression prevents timely degradation of (mRNA) *Notch1a* by the m6A ‘reader’ YTHDF2 (Zhang et al., 2017). A similar role for m6A has been observed during mouse HSC development (Lv et al., 2018).

With the integration of single-cell transcriptomics and epigenomics, increasing evidence has demonstrated that epigenetic regulation is indispensable during cell fate determination and conversion. Histone modifications, transcription factor binding, and three-dimensional genome structure data have recently been integrated to elucidate definitive hematopoietic development. These efforts revealed that regulatory regions associated with HSCs display active histone modifications as early as the AEC stage, preceding chromatin loop formation. Contrary to previous views, RUNX1 participates in enhancer-promoter (E-P) interactions beginning at the early AEC stage, where it forms transcriptional regulatory modules with other hematopoietic transcription factors to drive E-P interactions and gene expression throughout HSC ontogeny (Li et al., 2022). It was previously believed that HECs directly followed ECs in the developmental trajectory. However, Zhu et al. identified and characterized pre-HE cells between ECs and HECs using scRNA-seq. Further analysis with single-cell Assay for Transposase-Accessible Chromatin using sequencing (scATAC-seq) revealed that in pre-HE cells, a distal enhancer located 371 kb upstream of *Runx1* P1 promoter becomes accessible and gradually closes as HSC mature. Activation of this enhancer in pre-HE cells permits RUNX1 expression, enabling cells to overcome the bottleneck during transition to HECs. This process functions as an ‘epigenetic switch’ for the pre-HE cells to HECs transition (Zhu et al., 2020).

To determine the developmental stage at which HSPC heterogeneity is established, scRNA-seq and scATAC-seq were integrated to analyze endothelial and hematopoietic cells in zebrafish embryos at 36 hpf (hours post-fertilization). Analyses revealed that HSPCs already display distinct lineage preferences, with this diversity of lineage biases originating at the HEC stage. The transcription factor SPI2, a member of the E26 transformation-specific (ETS) family, was shown to contribute to the formation of lymphoid/myeloid-primed HSPCs from HECs but did not affect erythroid lineage formation (Xia et al., 2023). However, during hematopoietic development, it remains unclear which AECs are selected to specialize into HECs, whether this selection is random or specific, why these cells are chosen over others, and what molecular mechanisms underlie this process.

Emerging evidence highlights the roles of DNA modifications and post-transcriptional regulation in HSC emergence. scRNA-seq was used to identify approximately 7,000 long non-coding RNAs (lncRNAs), among which *H19* was found to be essential for EHT in the AGM region. *H19* functions through binding to and inhibiting S-adenosylhomocysteine hydrolase (SAHH), thereby promoting demethylation of several hematopoietic transcription factor promoters and facilitating EHT process (Zhou et al., 2019b). Single-cell full-length transcriptome analysis has revealed dynamic alterations in RNA alternative splicing (AS) during hematopoietic development. The number of transcript isoforms peaks at the type I pre-HSC stage and subsequently declines. The splicing factor SRSF2 was found to regulate the majority of these AS events. Its deficiency impairs HEC specification and disrupts the RNA splicing of critical factors such as RUNX1 and MYB, ultimately inhibiting HSC emergence, highlighting the importance of post-transcriptional regulation in hematopoietic development (Wang et al., 2022). The ongoing discovery of these epigenetic regulatory mechanisms has made the principles of HSC development increasingly complex and comprehensive. Future work is needed to determine whether these regulatory mechanisms are conserved or similar in human HSC emergence.

### 3.2 Precise identification and enrichment of distinct subpopulations during HSC development

Acquiring sufficient numbers of highly purified HECs, pre-HSCs, or HSCs remains a major challenge in the field of hematopoietic development. Based on previous knowledge, various marker combinations and transgenic fluorescent reporter lines have been utilized to isolate cell populations along the EHT. The primary strategies previously used for sorting ECs, HSC-generating precursors and HSCs are summarized in Table 1 (those not labeled with^a^).

**TABLE 1:** Enrichment strategies of distinct hematopoietic cell populations.

Cell populations	Enrichment strategies	References
EC	CD31^+^CD144^+^CD41^−^CD45^−^CD43^-^Ter119^−^	Zhou F. et al. (2016)
		Tang et al. (2021)
	CD31^+^CD144^+^CD41^−^CD45^−^Ter119^−^	Howell et al. (2021)
	CD144^+^CD41^−^CD45^-^Ter119^−^KIT^-^ *Gfi1:*tdTomato^−^ *Gfi1b:*GFP^−^	Fadlullah et al. (2022)
	CD144^+^CD41^−^CD45^-^Ter119^−^KIT^-^ACE^-^ *Gfi1:*GFP^−^ ^a^	
	CD144^+^CD44^low^KIT^−^ (AEC)^a^	Oatley et al. (2020)
	CD31^+^CD44^+^CD41^−^CD45^−^CD43^−^KIT^−^ (AEC)	Li C.C. et al. (2022)
Pre-HE cell	CD144^+^CD41^−^CD45^-^Ter119^−^KIT^-^ACE^+^ *Gfi1:*GFP^−a^	Fadlullah et al. (2022)
HEC	CD31^+^CD44^+^CD41^−^CD45^−^CD43^−^KIT^+^CD201^+^ (PK44)^a^	Hou et al. (2020)
	CD31^+^CD44^+^CD41^−^CD45^−^CD43^−^ *Neurl3*-EGFP^+a^	Hou et al. (2020)
	CD144^+^CD41^−^CD45^-^Ter119^−^KIT^-^ *Gfi1*:tdTomato^+^ *Gfi1b*:GFP^−^	Fadlullah et al. (2022)
	CD144^+^CD41^−^CD45^-^Ter119^−^KIT^-^ACE^+^ *Gfi1*:GFP^+^ ^a^	
Type I pre-HSC	CD31^+^CD41^low^CD45^−^KIT^+^CD201^hi^	Zhou F. et al. (2016)
	CD144^+^CD41^+^CD43^+^CD45^−^	Oatley et al. (2020), Kapeni et al. (2022)
	CD144^+^CD44^low^KIT^hi^ ^a^	Oatley et al. (2020)
Type II pre-HSC	CD31^+^CD45^+^KIT^+^CD201^hi^ ^a^	Zhou F. et al. (2016)
	CD144^+^CD41^+^CD43^+^CD45^+^	Oatley et al. (2020), Kapeni et al. (2022)
	CD144^+^CD44^hi^ ^a^	Oatley et al. (2020)
AGM HSC/HSPC	CD34^+^CD45^+^	Kapeni et al. (2022)
	CD31^hi^SSC^low^KIT^hi^GATA2^med^CD27^med^ ^a^	Vink et al. (2020)
	RUNX1^+^HOXA9^+^MLLT3^+^MECOM^+^HLF^+^SPINK2^+^ (human)^a^	Calvanese et al. (2022)
	CD144^−^CD41^+^	Oatley et al. (2020)
FL HSC	Lin^−^Sca-1^+^Mac-1^low^CD201^+^ (E12.0)	Zhou F. et al. (2016)
	CD45^+^CD201^+^CD48^−^CD150^+^ (E14.0)	Zhou F. et al. (2016)

^a^ newly established strategies; low, low expression; hi, high expression; med, medium expression.

scRNA-seq enables the resolution of cellular heterogeneity and molecular characterization of distinct cell subpopulations at single-cell level, allowing for the identification of molecular markers across diverse subsets. When combined with functional validation experiments, it significantly improves the enrichment efficiency of multiple hematopoietic cell subpopulations. Previous studies have identified CD201, also known as EPCR (Procr), as a surface marker of functional HSCs in the FL and BM (Balazs et al., 2006; Iwasaki et al., 2010; Benz et al., 2012). Zhou et al. demonstrated that CD201 facilitates the enrichment of type I pre-HSCs through OP9-DL1 co-culture and transplantation experiments. Subsequent scRNA-seq revealed that presumed CD31^+^CD45^+^CD41ˡᵒʷ type II pre-HSCs segregated into two clusters, of which only one highly expressed CD201. The use of CD31^+^CD45^+^KIT^+^CD201ʰ^i^ increased the enrichment efficiency of type II pre-HSCs by 2.1-fold (Zhou F. et al., 2016). CD44 expression was subsequently found to be heterogeneous in AGM aorta and gradually increased during EHT. When combined with KIT, CD44 enabled more precise separation of cell populations at distinct stages of EHT (Oatley et al., 2020). In the same year, Hou et al. performed scRNA-seq on E10.0–E11.0 AGM CD45^−^CD31^+^CD144^+^ cells, including ECs and CD41^+^ hematopoietic cells, which were classified into five subpopulations. CD44, CD201, and KIT were selected as candidate surface markers based on scRNA-seq data and subsequently validated for HEC enrichment through serial transplantation experiments. Subsequently, a novel combination, CD41^−^CD43^−^CD45^−^CD31^+^CD201^+^KIT^+^CD44^+^ (abbreviated as Procr^+^KIT^+^CD44^+^, PK44) was established for HEC enrichment. HECs isolated using this immunophenotype exhibited transcriptional profiles consistent with those inferred from computational analysis. Surprisingly, *in vitro* culture of single PK44 cells revealed that 2.7% possessed both endothelial and hematopoietic potential, indicating the existence of a rare but authentic population. *Neurl3* was found in further HEC marker gene screening, and a new fluorescent transgenic mouse line, *Neurl3*-EGFP, was generated, which can serve as an alternative to CD201 and KIT in enriching HSC-primed HECs (Hou et al., 2020).

Based on distinct strategies for cell populations sorting and varying sequencing depths, novel cell surface markers have also been identified through corresponding single-cell transcriptome analyses. The *Runx1b*-RFP and *Gfi1*-tdTomato/*Gfi1b*-GFP transgenic lines were utilized for scRNA-seq to distinguish subpopulations at different stages during HSC development. These analyses revealed that angiotensin-converting enzyme (ACE/CD143), although expressed at a relatively low level in AECs, is predominantly expressed in pre-HE cells and HECs. Functional validation confirmed ACE as a novel surface marker enriched in functional pre-HE cells and HECs (Fadlullah et al., 2022). *Gata2-*IRES-Venus reporter mice were employed to assess GATA2 expression levels. Combined with iterative single-cell sequencing and functional experiments, the combination of CD31^hi^SSC^low^KIT^hi^GATA2^med^

CD27^med^ enriched HSCs by 68.3-fold and further localized functional HSCs to IAHCs with small size, consisting of 1–2 cells in the ventral region (Vink et al., 2020).

HECs comprise a mixture of cells with differential hematopoietic potential. In a previous study, E10.0 AGM PK44 HECs were classified based on graph-based unsupervised clustering into three subgroups: endothelial-biased, hematopoietic-biased, and transitional. Indeed, these three subpopulations exhibit distinct molecular signatures. CD93, KIT, and CD146 have been identified as markers for further enrichment. Although not all HSC-primed HECs can be enriched, the KITʰ^i^CD93^low^ and KITʰ^i^CD146^low^ immunophenotypes enable a 1.3-fold and 1.7-fold enrichment, respectively (Li et al., 2021). Most current studies on cell enrichment focus on improving HEC enrichment strategies, and several novel markers or combinations have recently emerged. However, as only a subset of HECs ultimately generates HSCs, further understanding of the functional heterogeneity of HECs populations through single-cell multi-omics is crucial. Furthermore, identifying the molecular mechanisms regulating the differential hematopoietic potential of HECs is essential for guiding future *in vitro* functional HSC induction.

**FIGURE 4:**
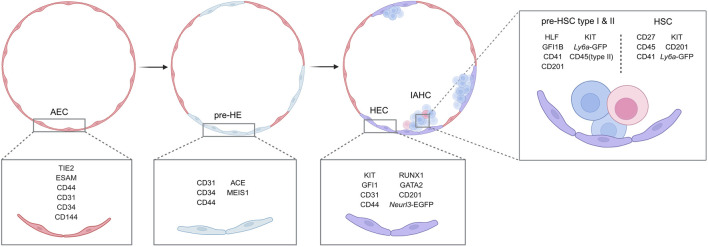
Dynamic gene expression patterns during EHT. The signatures defining key hierarchical developmental stages of hematopoietic stem cell (HSC) development in the aorta-gonad-mesonephros (AGM) region-including arterial endothelial cells (AECs), pre-hematopoietic (pre-HE) cells, hemogenic endothelial cells (HECs), pre-HSCs and HSCs-are summarized.

Based on these studies above, we have summarized the most recent sorting strategies for HSC-primed precursors and HSCs in Table 1, with the newly established strategies highlighted with^a^ for distinction. The significant variation in KIT expression levels observed among these populations, particularly within the HEC sorting strategies, likely reflects the intention to isolate HECs at different developmental states during the EHT. Furthermore, to visually represent the dynamic changes in cellular properties throughout HSC development, we summarize the current understanding of HSC generation in the mouse, including cell subpopulations, surface markers, and fluorescent transgenic reporter lines, in Figure 4.

### 3.3 Dissecting the microenvironment supporting HSC development

In addition to the aforementioned regulatory factors, the AGM microenvironment has been demonstrated to play a crucial role in HSC development. Among these, ECs are critical components of the AGM microenvironment. ECs serve as one of the primary sources of KITL, providing a critical microenvironment for the survival of type II pre-HSCs and HSCs in the AGM region, although type I pre-HSCs remain largely unaffected following endothelial-specific *Kitl* knockout (Azzoni et al., 2018). Mouse AGM-derived ECs with high AKT expression exhibit elevated levels of multiple Notch ligands, promoting the induction and expansion of transplantable HSCs from embryonic hemogenic precursors *in vitro* (Hadland et al., 2015). The subaortic mesenchyme is essential for hematopoietic cluster formation, and its absence significantly impairs this process, as demonstrated in chick embryos (Richard et al., 2013). Studies have also shown that AGM-derived stromal cell lines can serve as an *in vitro* microenvironment for HSC growth and maintenance (Oostendorp et al., 2002; Daniel et al., 2020). Another vital component of the AGM niche is the sympathetic nervous system, which facilitates HSC formation by promoting catecholamine secretion through GATA3 expression (Fitch et al., 2012). Furthermore, blood flow is also crucial for the emergence and maintenance of HSCs, mediated by nitric oxide (NO) and Yes-associated protein (YAP) signaling, respectively (North et al., 2009; Adamo et al., 2009; Lundin et al., 2020).

The application of single-cell sequencing technology has significantly enhanced our understanding of the hematopoietic microenvironment in the AGM region. Following their earlier work in 2012, Kapeni et al. discovered that *Cdkn1c* was highly expressed in AGM NGFR^+^ sympathoadrenal (SA) cells. Deletion of *Cdkn1c* increased the number of HSCs in the E11.0 AGM by amplifying catecholamine-secreting SA cells. To better understand the differentiation of neural crest, scRNA-seq analysis of NGFR^+^ cells in the AGM region was conducted and unexpectedly revealed that some neural crest cells transdifferentiate into mesenchymal cells after reaching the aorta, secreting HSC regulatory factors such as CXCL12 and BMP4. However, whether this cell population genuinely influences HSC development and its regulatory mechanisms remains unclear and requires further investigation (Fitch et al., 2012; Kapeni et al., 2022). It has also been reported that *Runx1* is expressed not only in HECs and hematopoietic cells but also in subaortic mesenchymal cells. scRNA-seq analysis of RUNX1^+^ subaortic mesenchymal cells revealed that they primarily consist of smooth muscle cells and PDGFRα^+^ mesenchymal cells. Pleiotrophin (PTN), which is expressed downstream of RUNX1 in PDGFRα^+^ mesenchymal cells, plays a crucial role in promoting EHT (Fadlullah et al., 2022). A subsequent study identified a population of NG2^+^RUNX1^+^ pericytes (PCs)/vascular smooth muscle cells (vSMCs) in the AGM region as a key niche component for HSC development. Deletion of *Runx1* in NG2^+^ cells significantly reduced the production of HSPCs from the AGM. The conditional knockout of *Runx1* broadly altered the gene expression profiles of both PCs/vSMCs and ECs, including genes related to extracellular matrix composition and vascular development, revealing the critical role of these niche cells in maintaining the AGM microenvironment (Gonzalez Galofre et al., 2024).

As a key component of the AGM microenvironment and also the source from which HSCs emerge, the regulation of ECs is often studied using *in vitro* culture systems. Also in a follow-up to a previous work, Hadland et al. conducted scRNA-seq on HSC-supportive, AGM-derived ECs and VE-Cadherin^+^CD61^+^EPCR^+^ (V^+^61^+^E^+^) HSC precursors. This analysis identified essential ligand-receptor interactions critical for HSC development and self-renewal, such as Notch signaling, VLA4-Fibronectin, and CXCL12-CXCR4. Based on these findings, a stromal cell-independent culture system was engineered, which successfully supported V^+^61^+^E^+^ cells isolated from E9.0-E11.0 embryos to generate HSCs with long-term transplantation capacity *in vitro* (Hadland et al., 2015; Hadland et al., 2022). Another study similarly highlighted the role of the vascular niche in HSC maturation. While E11.5 AGM pre-HSCs could be induced into functional HSCs with long-term transplantation capacity using only SCF and thrombopoietin (TPO) in serum-free conditions without feeder cells, E10.5 HECs and pre-HSCs required an additional endothelial microenvironment to mature into HSCs. This study reveals the essential role of the endothelial microenvironment for HSC development, although the reliance of HSCs on vasculature-derived factors is dynamic and varies with their maturation level (Morino-Koga et al., 2024). Vascular niche endothelial cells (VN-ECs), engineered from HUVECs transduced with the *E4orf1* gene, demonstrated the ability to support the conversion of AGM-derived hemogenic endothelial (aHE) cells and placenta-derived hemogenic endothelial (pHE) cells into HSPCs. However, HSPCs induced from pHE showed limited self-renewal capacity. A comparative analysis was conducted on the single-cell transcriptomes of the two types of HE-induced HSPCs described above, revealing significantly lower expression of self-renewal-related genes in pHE-derived HSPCs than in aHE-derived HSPCs. This downregulated expression pattern and the associated self-renewal capacity were enhanced by RA treatment (Liang et al., 2025).

The studies above significantly enhance our understanding of the microenvironment supporting HSC emergence *in vivo* (as summarized in Figure 3) and contribute to the optimization of culture conditions for *in vitro* HSC induction. However, since most current knowledge is derived from mouse models, it remains to be determined whether the cellular composition and key factors within the human AGM microenvironment align with those observed in mice. Furthermore, the AGM microenvironment comprises diverse niche cell types, including ECs, SA cells, and stromal cells. The spatial localization, interaction networks, and spatiotemporal dynamics of these cells within the niche remain poorly defined-yet such information is essential for accurately recapitulating the *in vivo* microenvironment *in vitro.*

## 4 Revealing human HSC ontogeny via single-cell multi-omics

Although model organisms such as mice and zebrafish have provided invaluable data for elucidating the process and regulatory mechanisms of mammalian HSC development, the direct study of human embryonic HSCs is essential for uncovering species-specific mechanisms and for better guiding the *in vitro* generation of HSCs from hPSCs. In recent years, the application of single-cell multi-omics technologies has significantly advanced our understanding of human HSC emergence, revealing the cellular heterogeneity and complex regulatory networks that orchestrate this process.

The application of single-cell multi-omics has enabled the construction of a comprehensive transcriptomic atlas spanning the entire process of HSC development. Recently, Zeng et al. established the first transcriptomic profile of the endothelial-to-hematopoietic transition across CS10-CS15 in human embryos. At CS12-CS14, they transcriptionally identified HSC-primed HECs characterized by an arterial endothelial signature (e.g., GJA5, GJA4, DLL4) and the co-expression of RUNX1, MYB and ANGPT1. Furthermore, this study identified a distinct population of HECs in human embryos appearing in CS10/11 earlier than the emergence of HSC-primed HECs (referred to as late-HECs) and thus termed early HECs. Compared to late-HECs, early HECs exhibit distinct molecular characteristics, notably lacking the expression of arterial markers, suggesting that they may not originate from arterial endothelium. However, the precise origin of early HECs, as well as their potential to contribute to full hematopoietic lineage differentiation like late-HECs, remains to be elucidated (Zeng et al., 2019). Complementing this work, a comprehensive developmental map of HSCs was constructed using human embryos from CS14 to birth. Subsequent UMAP analysis revealed a direct developmental trajectory from AECs to HSCs. This inference was further corroborated by spatial transcriptomics and immunofluorescence analyses, collectively supporting that human HSCs originate from AECs. In addition, it was demonstrated that HSCs can be distinguished with lineage-restricted progenitor cells using the combination RUNX1^+^HOXA9^+^MLLT3^+^MECOM^+^HLF^+^SPINK2^+^. The study further elucidated HSC maturation in the FL and defined stage-specific markers for HSCs from emergence to maturity. Additionally, HSCs were detected in both the placenta and YS (Calvanese et al., 2022).

Single-cell omics was recently employed to characterize the molecular signatures of human HECs and to guide strategies for their enrichment. In humans, CD44 was identified as a marker distinguishing human AECs and CD44-based enrichment increased HEC isolation efficiency by at least 10-fold (Zeng et al., 2019). ACE colocalized with RUNX1 in the ventral wall of the aorta at CS12, coinciding with the emergence of the first hematopoietic cell cluster, and CD34^+^CD45^−^ACE^+^ cells express genes associated with arteries and HECs. CD32 is one of the genes enriched in ACE^+^ cells, and subsequent studies identified it as a marker for enriching functional human HECs from embryo and hPSC differentiating cultures. Moreover, scRNA-seq of day 8 Wnt-dependent (Wntd) CD34^+^CD43^−^CD184^−^CD73^−^ cells showed that CD32^+^ HECs occupy an intermediate state among six HEC subpopulations and CD32 expression indicates the transition from Notch-dependent to Notch-independent states (Scarfò et al., 2024).

While the regulation of HSC emergence has been extensively studied in model organisms like mice and zebrafish, the mechanisms governing this process in humans remain less explored. Recently, Crosse et al. utilized spatial transcriptomics and scRNA-seq to identify the secretory factors required for human HSC emergence. They discovered that the cardiac EGF signaling pathway is enriched in the ventral domain of the dorsal aorta. As a key regulator of this pathway, Endothelin-1 (EDN1), produced primarily by AECs, was found to be enriched near ventral IAHCs and to promote hematopoietic progenitor formation in human embryonic stem cell (hESC) culture system, highlighting its role as a novel critical regulator of the human HSC niche (Crosse et al., 2020). In a subsequent study, Crosse et al. further integrated spatial transcriptomic data spanning the entire HSC emergence window (CS13 to CS17) with scRNA-seq data covering CS13 to CS15, revealed that the birth of definitive HSCs is accompanied by a dramatic ventral polarization and increased complexity of signaling pathways in the AGM region. Key pathways upregulated during this process included pro-inflammatory signaling (e.g., TNFα/NF-κB, IL6/JAK/STAT3), epithelial-mesenchymal transition (EMT), TGF-β signaling, and hypoxia. Additionally, dynamic expression of adhesion molecules and *HOX* genes was observed during HSC emergence, though the precise relationship between these dynamic expression patterns and HSC generation requires further investigation in the future (Crosse et al., 2023). Furthermore, the role of RA signaling in regulating HEC generation from hPSCs was investigated by Luff et al. They identified two distinct hematopoietic pathways derived from Wntd mesoderm, which differ in their RA dependence. The RA-dependent HECs exhibited a transcriptional profile more similar to embryonic HSC-competent HEC. In contrast, the RA-independent HE resembled non-arterial early HECs (Luff et al., 2022). Collectively, these studies provide new insights for exploring the regulatory mechanisms underlying human HSC ontogeny. However, how to orchestrate these mechanisms to faithfully recapitulate the entire HSC developmental process from hPSCs *in vitro* remains a major challenge.

## 5 Discussion and future perspectives

The application of single-cell omics technologies has greatly advanced our understanding of HSC origins. The developmental journey of HSCs has been revealed as a complex, multi-stage process characterized by cellular heterogeneity, precise regulation of diverse transcription factors, signaling pathways, and epigenetic mechanisms, as well as interactions with specialized microenvironments. In this review, we summarize how single-cell studies have illuminated the developmental processes and regulatory mechanisms of embryonic HSCs. Although considerable advances have been made, a number of unresolved challenges and unknowns await future research.

Given the relative scarcity of accessible human embryonic samples, mouse and zebrafish models have become indispensable in hematopoietic research, yielding a wealth of high-resolution single-cell transcriptomic data. In contrast, single-cell developmental data of HSC origin derived from human embryonic samples are still scarce. In the future, integrating published data to conduct in-depth integrated analyses of single-cell data during HSC development in mice, zebrafish and humans, combined with rigorous functional experimental validation, will be crucial for further understanding the mechanisms underlying HSC development *in vivo* and guiding HSC induction *in vitro*.

Previous studies have noted that HSCs emerge predominantly from the ventral wall of the dorsal aorta, with far fewer derived from the dorsal region, a pattern driven by a complex interplay between dorsally- and ventrally-derived signals, including SHH, KIT, and BMP pathways (Richard et al., 2013; Chandrakanthan et al., 2022; Yvernogeau et al., 2023; Souilhol et al., 2016). Further studies have revealed that the middle segment of the dorsal aorta exhibits a significantly greater capacity to generate HSCs than its terminal regions, potentially associated with blood flow (Yokomizo and Dzierzak, 2010; Mascarenhas et al., 2009). These studies collectively demonstrate an uneven spatial distribution of HSC emergence along the aorta. This regional variation may be associated with endothelial heterogeneity and distinct stromal microenvironments surrounding different vascular segments. With the advancement of single-cell spatial omics, it is now feasible to further dissect the cellular heterogeneity of the aorta and its surrounding niche at the three-dimensional level, which may provide additional insights into HEC specification and the process of EHT.

Multiple niche cell types, including vascular endothelial cells, stromal cells, and neural crest cells, contribute to the composition of the AGM microenvironment (Chandrakanthan et al., 2022; Hadland et al., 2022; Kapeni et al., 2022). However, whether additional microenvironmental cell types that are as yet unidentified exist remains a critical question. The application of high-throughput, unbiased scRNA-seq to identify potential additional microenvironmental cell types in the AGM and to explore their roles in regulating HSC development will be crucial for optimizing *in vitro* HSC induction strategies. In addition, AGM microenvironmental cells exhibit distinct spatial distribution patterns. Mimicking the cellular organization and hemodynamic environment of the native AGM niche to construct AGM-like organoids *in vitro* may help overcome current challenges in HSC induction.

HSC transplantation has been successfully applied to treat a wide range of hematologic and non-hematologic diseases. However, its broader application is constrained by limitations such as donor scarcity, inefficient *in vitro* expansion of HSCs, and risks associated with transplantation-related complications and mortality (Grinstein and Mahotka, 2023). The induction of transplantable HSCs from hPSCs *in vitro* represents a highly promising direction for future research. Although previously reported hPSC-derived HSC-like cells closely resemble natural HSCs, some gaps still remain in both molecular characteristics and functional properties, particularly in hematopoietic potential and engraftment efficacy (Sugimura et al., 2017). Recent work by Piau et al. demonstrated the successful generation of long-term reconstituting, multilineage HSCs from hPSCs under vector-free and stroma-free conditions. However, their approach faces challenges in elucidating the regulatory mechanisms provided by the embryoid body (EB) microenvironment during HSC formation. Moreover, achieving the requisite cell numbers and purity for clinical transplantation remains a significant hurdle (Piau et al., 2023). In a subsequent study, Ng et al. also generated HSCs with multilineage potential using a defined medium supplemented with RA. Despite this advancement, their protocol still encounters limitations including low generation efficiency, insufficient self-renewal capacity, and limited secondary transplantation potential (Ng et al., 2023). These studies underscore that the conditions for the *in vitro* generation of HSCs from hPSCs necessitate further optimization. A continuous scRNA-seq analysis of the entire *in vitro* HSC induction process from hPSCs is currently lacking. To further elucidate this process, comprehensive single-cell profiling of the entire *in vitro* induction trajectory, compared with *in vivo* HSC development, could clarify why existing induction systems remain limited.

The generation of functional blood cells from hPSCs by mimicking *in vivo* hematopoiesis offers a promising path to a safe and sufficient blood supply with reduced alloimmunization risks (Lapillonne et al., 2010; Suzuki et al., 2020; Ackermann et al., 2018). As HSCs serve as a critical intermediary in this process, the inefficient derivation of long-term HSCs represents a key obstacle in this field (Ebrahimi et al., 2020; Hansen et al., 2019). Recent single-cell multi-omics analyses have refreshed our knowledge by uncovering the heterogeneity and complex regulatory mechanisms of *in vivo* HSC development. The integration of these findings is crucial for refining *in vitro* differentiation protocols and ultimately enhancing the output of therapeutic hematopoietic lineages.

Altogether, advances in single-cell omics technologies have greatly enhanced our understanding of HSC development. Nevertheless, many aspects remain unexplored, and their resolution may pave the way for future clinical applications of *in vitro* HSC induction.
